# The European Council of Legal and Forensic Medicine: its role and work in progressing harmonised standards and recommendations in Forensic and Legal Medicine investigation procedures, training and teaching through pursuit of internationally agreed and peer-reviewed published guidelines

**DOI:** 10.1007/s00414-025-03446-w

**Published:** 2025-02-22

**Authors:** Denis A. Cusack

**Affiliations:** 1 European Council of Legal and Forensic Medicine (ECLFM), Dublin, Ireland; 2 Coroner Service, Dublin, Ireland; 3 Medical Bureau of Road Safety, School of Medicine, University College Dublin, Dublin, Ireland; 4 RCSI and UCD Malaysia Campus, George Town, Penang Malaysia

**Keywords:** ECLFM, Harmonised standards, Forensic and legal medicine investigation procedures, Published guidelines

## Abstract

Specialists and Practitioners in Forensic Medicine and Pathology have onerous duties and obligations in the examination of both the dead and living victims of alleged criminal assault and injury and in the examination of the alleged perpetrators of criminal actions. Forensic physicians and pathologists work in close collaboration with scientific colleagues and law enforcement agencies and present their independent and unbiased findings to the criminal, civil and coronial courts to assist in the administration of justice. Approved and established forensic standards for reporting and certifying investigations and conclusions are core prerequisites for the maintenance of evidential credibility and scrutiny and of justice. Judges, Investigating Magistrates, Coroners and Prosecution and Defence Lawyers as well as Government Departments of Justice and Health are recipients of such expert reports and testimony in framing their respective tasks for society within their countries’ legal frameworks. The European Council of Legal and Forensic Medicine has published collaborative papers on international standards and guidelines for forensic examinations by forensic physicians and pathologists of deaths and injuries in the areas of accreditation of pathology services; on-site scene and corpse investigation; examination of victims of sexual assault and of elder abuse; and also in undergraduate and postgraduate forensic teaching and training, in pursuit of international harmonisation.

## Introduction

The European Council of Legal and Forensic Medicine (ECLFM, formerly the European Council of Legal Medicine, ECLM) was founded in 1992 and is the official body dealing with matters relating to the discipline of Legal and Forensic Medicine in Europe. It deals with all scientific, educational and professional matters pertaining to the discipline on a European level. The Delegates to the ECLFM are drawn from 36 countries across Europe and comprise persons professing to be specialists in the discipline of Forensic and Legal Medicine who are medically qualified and involved in the investigation, evaluation and education of unexpected and/or unnatural deaths and bodily harm within the framework of the legal system and/or in the teaching of the specialty [1].

There are many national, regional and international specialist bodies throughout the world responsible for studying and issuing guidelines on forensic procedures. The ECLFM does not claim or have a monopoly on the harmonisation process but it constitutes a large bloc of countries working together to achieve those harmonised standards.

The Specialty encompasses a number of distinct areas of medical practice in assessment of both living and dead persons for the purpose of investigating assault and injuries in the context of criminal actions and human rights and of investigating death within the legal framework of the particular jurisdiction in which the events occur. These events include examination of the victims of physical and sexual assault and abuse and of perpetrators. They also include the mental health assessment of persons detained or in custody, assessment of intoxicated drivers and the examination of scenes of death and of mass disasters where partial or whole human remains are found and the examination of the bodies or partial remains.

The forensic medical experts practice primarily in the subspecialties of forensic pathology and clinical forensic medicine. The forensic continuum in which they practice comprises the authoritative governing laws of the territorial jurisdiction as enacted by the particular legal body which in turn are informed by forensic medicine and science. These laws and principles feed into how police enforcement and investigation are performed and then lead into analysis by forensic pathology and forensic laboratories. The information derived may then proceed into a prosecutorial, civil or inquisitorial judicial process leading to a judicial decision and outcome.

Between 2014 and 2024 the partnerships within the European Council of Legal and Forensic Medicine (ECLFM, formerly the ECLM) through the publication of peer-reviewed guidelines for forensic investigative procedures have facilitated the publication of six papers ranging from accreditation of forensic pathology services in Europe to guidelines for the on-site examination of dead persons and of victims of sexual assault and elder abuse. It has also resulted in a published review of undergraduate medical education across Europe in the area of Forensic and Legal medicine.

## International guidelines and standards in forensic death investigation

### Forensic pathology services accreditation

The ECLM published its guidelines for accreditation of forensic pathology services in Europe in 2015 [2]. They emphasised the need for fundamental standards for the services provided in the justice and healthcare systems to be independent, effective and prompt with access to appropriate facilities and performed by independently certified and accredited processes. These accreditation criteria applied to forensic services in their policies, procedures and facilities rather than individual practitioners who would be dealt with under separate guidelines. They were also set as the minimum standards to attain recognition for reliable pathology services and were set out in 9 pages of detailed accreditation standards under 8 main headings: General (Facilities, Mass Disaster Plan etc.); Investigation (Scene investigation, Identification etc.); Morgue Operations (Autopsy room, Radiology, Specimen collection); Histology; Toxicology; Reports and Records; Forensic Pathologists and Other Staff; and Support Services and Consultants. The accreditation process and protocols underpin harmonisation of standards ensuring a consistency for the investigating and adjudicating authorities to be able to rely on the Forensic Doctor’s and Scientist’s work.

The ECLM document on Harmonisation of Medico-Legal Autopsy Rules, published by the ECLM in 1994 and updated in 2014, formed the basis for the aforementioned subsequent publication and was also adopted by the Council of Europe in its Recommendation Nº R (99) 3 of the Committee of Ministers to Member States in 1999 on the Harmonization of Autopsy Rules [3, 4].

### Guidelines for on-site investigation

In 2017 the ECLM published its principles for on-site forensic and medico-legal scene and corpse investigation [5]. These included: Initial assessment on arrival at site; Viewing and examining the body and site; Collection and preservation of relevant evidence (including relevant collateral information); Consequential matters; and Materials and equipment. These principles are to be followed whenever a medical expert is required to perform an on-site corpse inspection to be utilized as general guidelines but adapted to the specific situation and interpreted using common sense and scientific knowledge of the relevant procedures and facts of the case. This should ensure that a comprehensive and professional report is available to the judicial authority (investigating judge, magistrate or coroner) in the relevant judicial and legal system. This on-site inspection by the forensic practitioner is a mandatory and essential stage of the forensic and medico-legal autopsy providing critical information for the subsequent investigation stages.

### Guidelines for on-site inspection forms

The general on-site inspection guidelines were followed by the 2022 publication of the ECLM guidelines for on-site Inspection Forms covering forensic pathology, anthropology, odontology, genetics, entomology and toxicology [6]. The purpose of these guidelines was to harmonise the procedures for a correct search, detection, collection, sampling and storage of all elements which might be useful as evidence and to ensure documentation of all these steps. The inspection forms provide a checklist to be used on-site at a crime or suspicious death scene. It permits the collection of all relevant data and materials for all of the various forensic specialists involved in the investigation thus supporting a collaborative work approach. Detailed instructions for the completion of the forms are included in the published paper.

## International guidelines and standards in forensic examination of the living victim of assault and alleged perpetrator

### Guidelines for examinations in sexual and physical assaults

Forensic clinical examination of the living victims of assaults were considered in the 2018 ECLM publication on harmonised examination of victims of sexual assault [7]. This is an area of complexity with medical, psychological and legal aspects. The guidelines set out general principles; the medical examination (including the taking of the history); collection of samples; the medical report; and the particular issue of victims who are minors. Forensic experts play a major role in the forensic and gynaecological medical examination and evidence in order to ensure the chain of custody is maintained. Victims should be examined by a specially trained medico-legal and forensic examiner in surroundings which meet the minimum healthcare standards. Treatment of a sexual assault victim is time-intensive and should be provided by a team which includes a forensic doctor. The guidelines can be used as the basis for a day-to-day service document or as a guide to develop further the health service for such patients.

### Guidelines for the examination of suspected elder abuse

The ECLM then followed with a second publication on living victims in 2019 with the issuing of guidelines in the increasingly recognised area of suspected elder abuse [8]. The paper presented a background in human rights (including Article 25 of the Charter of Fundamental Rights of the European Union) and outlined the types of abuse encountered. The importance of respect for the rights of older persons to lead a life of dignity and independence and to participate in social and cultural life was highlighted. It explored the prevention and management of such abuse and the family and institutional settings in which it occurs together with the importance of early detection. The guidelines summarised the examination of the elder person and the contents of the forensic medical report.

### Further guidelines being developed

Guidelines for the examination of victims of child abuse are now being considered by the ECLFM together with areas of humanitarian forensic medicine in mass disaster and migration.

## Forensic and legal medicine education and training

### Postgraduate specialisation

One of the most important issues to arise for any medical specialty is the education and training of specialists practising in the field to ensure the future continuation of competence and high standards of practice in a sufficient number of practitioners for the duties and tasks required. In 2011 the ECLM Delegates approved at its 11th General Assembly a seminal document on *Description of Legal and Forensic Medicine as a Medical Specialty in the EU* with the aims and objectives for Specialist Training in Legal and Forensic Medicine [9] =. This formed the basis for the submission in 2013 to the Union Européenne des Médicins Spécialistes (European Union of Medical Specialists, UEMS) by the ECLM Executive Board of the European Council of Legal Medicine for and on behalf of the Member States of the ECLM. The UEMS organisational objective is to improve patient care throughout Europe by developing and supporting excellence in specialist medical practice through the study, promotion and harmonisation of the highest level of medical specialists, medical practice and health care within the European Union. UEMS activities and projects are concrete means to serve medical specialists in EU to aim at this objective [10]. The Thematic Federation in Legal and Forensic Medicine was consequentially founded as a structure within the UEMS in 2013 and is currently being reviewed collegially by the ECLFM and the UEMS in the context of Monospecialty recognition within the EU.

The ECLM description includes an analysis of the specialty profile; entry criteria for the specialisation; core competencies and knowledge in clinical forensic medicine, thanatology, forensic pathology and traumatology; and medical law, ethics and related jurisprudence. It sets out the developed and advanced knowledge, experience and skills expected of the specialist together with other professional aspects of the specialist training programme, management skills and training, education and teaching, quality assurance. The Specialist Trainee must participate in both clinical and basic research leading to a higher degree or qualification. The training and the timeframe for this specialist training should be a minimum of four years full-time work with this and the time spent in practical experience being in accordance with the individual countries’ stipulations.

### Undergraduate teaching in forensic and legal medicine

In common with all medical specialties, undergraduate training of medical students provides a broad programme in the basic medical sciences and clinical training across all major cognate areas, there is a need to ensure that the future doctor in practice possesses the basic skills to deliver the core elements of healthcare to their future patients. In this Forensic and Legal Medicine is no different as all medical practitioners throughout the lifetime of their professional practice require basic knowledge of death certification; medical law and ethics; recognition and diagnosis of wounds; intoxicated driving; medico-legal report writing; and toxicology and prescription writing to name but a few examples. To round off its consideration of the specialty, the ECLM undertook a project to review the provision of teaching in the subject across its Member States in Europe. This resulted in the publication in 2024 on the current status of undergraduate teaching across Europe [11]. The review was carried out by means of a survey and the responses showed considerable emphasis on undergraduate teaching of forensic medicine in all countries with the exception of Belgium and the United Kingdom. There was great general consistency in the subjects taught. The data from this survey provide a baseline which should assist in developing a strategy to harmonise forensic and legal medicine undergraduate training in member countries of the ECLFM. The ECLFM is now in a good position to establish a pan-European working group to coordinate a consensus document identifying an appropriate and modern core undergraduate forensic medicine curriculum updating the ECLM Perugia document of 1992 [12]. This can then be presented to the medical education authorities in each country, and can be adapted for local requirements, based on available personnel, the forensic medicine structure in the country, and most importantly, the needs of the local population.

## Conclusions on the role and work of the European Council of Legal and Forensic Medicine

Through the collaborative work of Forensic and Legal Medicine Specialists from Europe and wider international regions, Forensic Medicine and Science are translating from the scene and bench to application and contributions to forensic investigation, the courts and justice systems and health and epidemiology. Forensic and Legal Medicine is a medical specialty in daily practice and action for justice and health of citizens through combining, integrating and harmonizing professional standards in accordance with best international practice protocols and guidelines. A strong specialty of Forensic and Legal Medicine in all jurisdictions adapted to meet local jurisdictional requirements is always an independent and forceful guardian of the rights of the most vulnerable in our societies in the practice overlap across medicine, science, law and justice.

The ECLFM continues to work on further harmonization standards such as in the area of child abuse and in developing educational standards in undergraduate and postgraduate forensic teaching. It also strives to conclude agreement in the wider recognition of the specialty as a monospecialty for training programmes and medical certification across Europe and internationally. The ongoing promotion of Forensic and Legal Medicine in the public good will continue to be the focus for the transnational work of the ECLFM.

### Future projects for the ECLFM

The European Council of Legal and Forensic Medicine repeats that it does not claim to have any monopoly on the practice and standards in Forensic and Legal Medicine and acknowledges the worthy, valuable, learned and comprehensive contributions of other National and International Bodies in the particular specialty and its subspecialties. It is however an organization now representing 36 countries within Europe (the 27 Members States of the EU and 9 other European countries) who have nominated Delegates to further the aim working together to achieve harmonised standards in Forensic and Legal Medicine in the pursuit of justice and healthcare for all citizens within Europe and wider afield for citizens of the world where justice and healthcare are increasingly transnational and global. The ECLFM current and future projects will continue with that purpose to the fore.

## Data Availability

Data sharing is not applicable to this article as no datasets were generated or analysed during the current study.
